# Regression of fibrous dysplasia in response to denosumab therapy: A report of two cases

**DOI:** 10.1016/j.bonr.2021.101058

**Published:** 2021-04-09

**Authors:** Maartje E. Meier, Wouter van der Bruggen, Michiel A.J. van de Sande, Natasha M. Appelman-Dijkstra

**Affiliations:** a Department of Orthopedic Surgery, Center for Bone Quality, Leiden University Medical Center (LUMC), Albinusdreef 2, 2333ZA Leiden, the Netherlands; b Section of Nuclear Medicine, Department of Radiology, LUMC, Albinusdreef 2, 2333ZA Leiden, the Netherlands; c Department of Nuclear Medicine, Slingeland Hospital, Kruisbergseweg 25, 7009 BL Doetinchem, the Netherlands; d Department of Internal Medicine, Division of Endocrinology, Center for Bone Quality, LUMC, Albinusdreef 2, 2333ZA Leiden, the Netherlands

**Keywords:** Fibrous dysplasia, McCune Albright syndrome, Denosumab, Antiresorptive, Nuclear imaging

## Abstract

We present two patients with fibrous dysplasia who showed a decrease in lesional size and activity after denosumab therapy. Both patients also experienced a reduction in pain and bone turnover markers, which had not been accomplished during previous bisphosphonate therapy. These cases highlight the potential of denosumab to decrease lesional size in fibrous dysplasia. This finding has been reported in mice, but not in humans. Denosumab may be considered when bisphosphonates are not tolerated or not effective (enough), or in severe cases as neoadjuvant therapy to improve surgical possibilities and outcome. In addition, these results show that Na[^18^*F*]F PET-CT is suitable for detecting change in each fibrous dysplasia lesion distinctively.

## 1 Introduction

Fibrous dysplasia (FD) is a rare skeletal disorder caused by a postzygotic mutation in the *GNAS* gene (Weinstein et al., 1991). FD can present as a solitary lesion in monostotic FD or as polyostotic disease with lesions in multiple bones. McCune Albright syndrome (MAS) is established when FD coincides with endocrinopathies or with other extra skeletal features such as *GNAS-*positive tumors or café au lait patches, while FD with intramuscular myxomas is identified as Mazabraud syndrome. In FD/MAS (OMIM#174800) fibro-osseous skeletal lesions are formed, inducing a variable, potentially severe clinical presentation that can include pain, fractures and deformities (Boyce and Collins, 2019; Hartley et al., 2019). The *GNAS* mutation disrupts the maturation of mutated osteoprogenitor cells into osteoblasts and thus bone formation. Bone remodeling is further disturbed by the increased production of cytokines including IL-6 and RANKL, stimulating bone resorption (Boyce and Collins, 2019; De Castro et al., 2019). RANKL is enhanced locally in FD tissue and in serum of patients (De Castro et al., 2019). In addition overproduction of fibroblast growth factor (FGF) 23 by osteoprogenitor cells can lead to elevated serum levels of FGF-23 and to hypophosphatemia due to renal phosphate wasting, which occurs in 50% of patients and further impairs bone mineralization (Collins et al., 2001). The physiology of bone formation, remodeling and metabolism can be visualized adequately and objectively by Na[^18^*F*]F PET-CT, and the advantages of this method over bone scintigraphy have recently been established for the assessment of FD/MAS (Papadakis et al., 2019; van der Bruggen et al., 2020). Symptomatic FD can be treated with surgery or antiresorptive treatment, mainly bisphosphonates, which have been used frequently but do not alter lesional uptake or growth and may not always be effective enough to reduce pain (Javaid et al., 2019; Rotman et al., 2019; Majoor et al., 2017; Chapurlat et al., 2004; Chapurlat et al., 1997). In bisphosphonate-resistant patients, patients who still have complaints of pain, elevated bone turnover markers and active lesions on imaging after adequate bisphosphonate therapy, the monoclonal antibody denosumab (Dmab) might be a promising alternative (Majoor et al., 2019). This RANKL-inhibitor suppresses osteoclastogenesis and has demonstrated to halt lesional growth, prevent lesion formation and induce mineralization in a FD/MAS mouse model (Palmisano et al., 2019). In humans only a slower growth rate has been described in one child with MAS treated with denosumab (Boyce et al., 2012), but not regression of lesions and not in adults. We here present two patients who showed a decrease in lesional size and activity with an excellent clinical and biochemical response after Dmab therapy. Both patients provided informed consent for the publication of their data.

## 2 Case report

### 2.1 Case 1

**Fig. 1:**
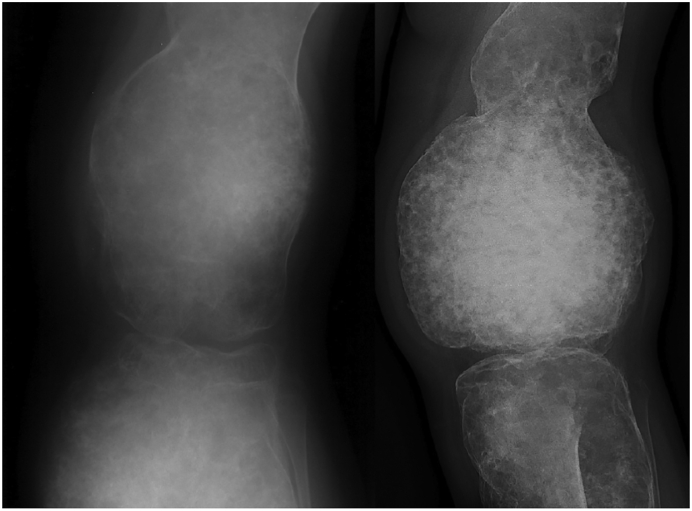
Progression of the lesion in the distal femur of case 1 prior to denosumab therapy on plain X-ray. Left: 1994. Right: 2015. Measurements could not be compared because left X-ray was not calibrated.

A 15-year-old girl was referred to our outpatient clinic with PFD affecting the left pelvis, femur, tibia, fibula, talus, navicular bone, cuneiform bones and first ray of the foot (skeletal burden score (Collins et al., 2005) 16.8). Diagnosed at age 5, she suffered from pain, leg length discrepancy, severe deformity, restricted mobility and multiple fractures since young age. At age 6 a bone allograft was transplanted in the hip and at age 24 a debulking procedure of the tibia was performed with inlay allograft reconstruction. Both procedures provided no substantial benefit. The lesions progressed over time (Fig. 1), while exacerbating pain, tiredness, swelling and impaired ambulation despite a brace.

**Fig. 2:**
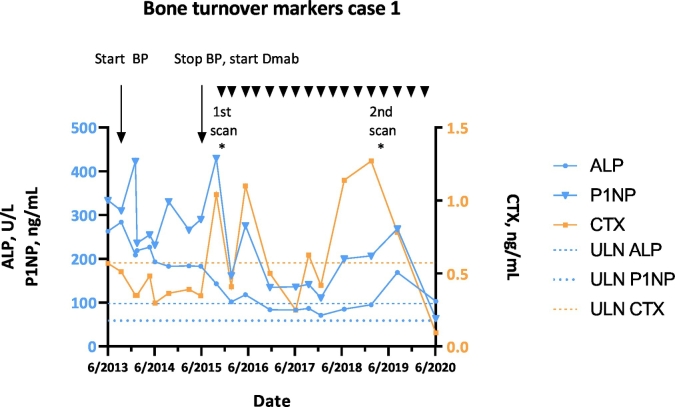
Bone turnover markers ALP, P1NP and CTX before and during bisphosphonate (BP) and denosumab (Dmab) treatment of case 1. Arrowheads indicate the denosumab injections. ULN: upper limit of normal.

Additionally the markers of bone turnover (BTMs) alkaline phosphatase (ALP), procollagen 1 N-terminal propeptide (P1NP) and beta-crosslaps (CTX) were consistently elevated. Lesional uptake on bone scintigraphy was markedly increased. At age 35 suppletion of calcium-vitamin D3 with bisphosphonate therapy was started, when calcium was 2.30 mmol/L (normal 2.15–2.55), phosphate 0.86 mmol/L (0.90–1.50), ALP 263 U/L (<98), P1NP 334 ng/mL (<59), CTX 0.570 ng/mL (<0.573) ng/mL and FGF-23135 RU/mL (<125) (Fig. 2). Six olpadronate infusions of 8–12 mg each were given 3-monthly with oral olpadronate 200 mg/day in between. Despite 1.7 years of treatment with a cumulative intravenous dose of 68 mg and oral dose of 96.000 mg, neither pain reduction nor normalization of BTMs were acquired, leading to opioid use and the maximum recommended dose of acetaminophen and diclofenac. Pain scores rose till 9 out of 10 for maximum pain and 8/10 for average pain. At age 37 denosumab (Prolia®, Amgen Europe B.V.) 60 mg was started subcutaneously every 3 months after informed consent for the off-label use. Calcium was 2.34 mmol/L, phosphate 0.86 mmol/L, ALP 183 U/L, P1NP 290 ng/mL, CTX 0.347 ng/mL and FGF-23132 RU/mL (Fig. 2). After several injections, the pain declined. The patient could taper off the opioids and could eventually quit all analgesics. Most recent pain scores were 5 for maximum and 5 for average pain, a substantial improvement. Denosumab enabled her to walk longer and farther and even without brace for the first time in her life. ALP decreased after 3 months to 143 U/L and normalized after 17 months. P1NP declined to half of pretreatment levels. CTX levels were normal before the start of denosumab, presumably as a result of the pretreatment with bisphosphonates, and varied during treatment because of a rebound peak right before the administration of the next dose (Fig. 2).

**Fig. 3:**
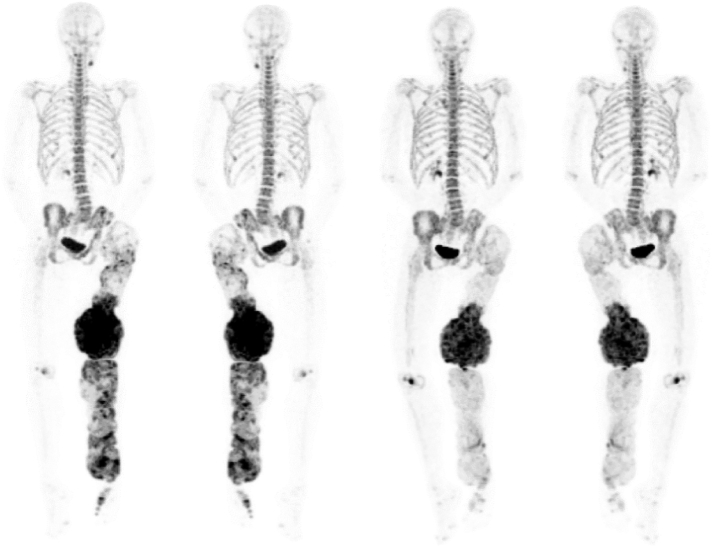
Na[^18^*F*]F PET-CT of case 1 before denosumab treatment (left, anteriorand posterior view) and after almost 4 years of therapy (right, anterior and posterior view). At the second scan, SUVpeak was decreased by 55%, SUVmean by 14%, FTV by 61% and TLF by 66%.

However the most striking results were observed on Na[^18^*F*]F PET-CT scan: after 14 months of treatment, the SUV (standardized uptake value, the tissue activity concentration normalized for the injected agent per unit bodyweight) peak declined by 55%, the SUVmean by 14%, FTV (Fluoride Tumor Volume) by 61% and TLF (Total Lesion Fluorination, based on lesional FTV and SUVmean) by 66% (Fig. 3).

**Fig. 4:**
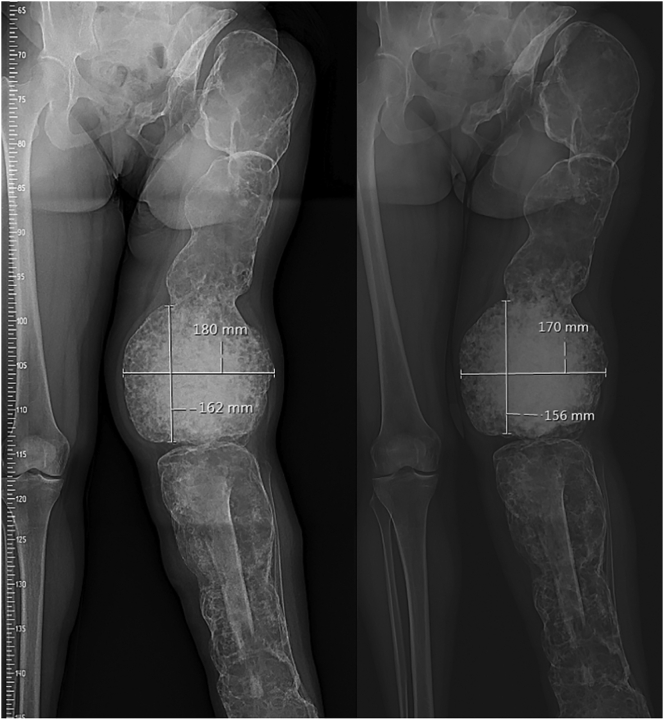
Regression of the lesions in the distal femur before (left) and after 3 years of dmab therapy (right) on plain X-ray of case 1. In the tibia, the bone allograft is visible which was transplanted during previous surgery.

The largest lesion in the distal femur decreased from 18.0 × 16.2 cm to 17.0 × 15.6 cm (Fig. 4). The Hounsfield Units (HUs) of the CT ranged between 10 and 1230, indicating a combination of fluid-filled cystic/lytic components, fibrous tissue, mineralized trabecular bone and cortical bone. The lesion regression was induced by a reduction in volume of the fluid-filled cystic components with HU between approximately 10 and 40 and of the fibrous tissue (HU 40–250). In addition to a reduction in volume, increased sclerosis indication mineralization and thus treatment effect was observed on the Na[^18^*F*]F PET-CT and on plain radiographs.

The patient has now used denosumab for 5.5 years without adverse effects.

### 2.2 Case 2

**Fig. 5:**
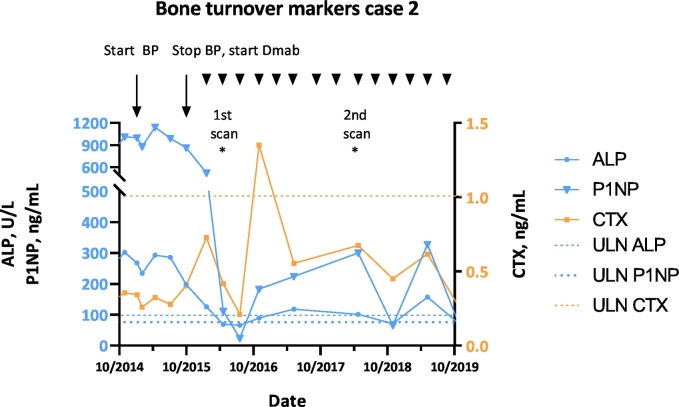
Bone turnover markers ALP, P1NP and CTX before and during bisphosphonate (BP) and denosumab (Dmab) treatment of case 2. Arrowheads indicate the denosumab injections. ULN: upper limit of normal.

The second patient had not experienced debilitating complaints until age 57, when a stabbing pain at the right side of her back appeared, present at rest, exacerbating with motion and progressive over time. In addition, she felt dyspnoic and had suffered from multiple pneumonias. Physical examination revealed swelling and deformity of the ribs and scoliosis, and a severely limited restrictive pulmonary function was found. Radiographical evaluation lead to the diagnosis of Mazabraud syndrome at age 61, with FD lesions located in the thoracic spine and multiple ribs bilaterally (SBS 2.7), combined with a myxoma in the right quadratus lumborum muscle. The lesions were expansile and accounted for the volume loss of the right hemithorax. Surgical treatment of the extensive rib lesions was relinquished due to the high risk for complications at this location, so bisphosphonate therapy and suppletion of calcium-vitamin D3 were started at age 67. The patient received 3 olpadronate infusions in a cumulative dose of 72 mg. Before therapy ALP was 302 U/L, P1NP 1012 ng/mL and CTX 0.356 ng/mL, afterwards ALP was 286 U/L, P1NP 990 ng/mL and CTX 0.278 ng/mL This treatment reduced the complaints only partially, but not sufficient. Therefore denosumab 60 mg every 3 months was started at age 68. During the first few months the pain was stable (maximum pain 7/10, average 6/10), but after 2 injections the pain declined. After 4 years of treatment maximum pain was 3 and average pain 0. In addition she could undertake more activities. Likewise the BTMs responded well: P1NP and ALP decreased to 1/3 of the pretreatment values and ALP reached near-normal levels. Again CTX was low pretreatment and fluctuated during treatment (Fig. 5).

**Fig. 6:**
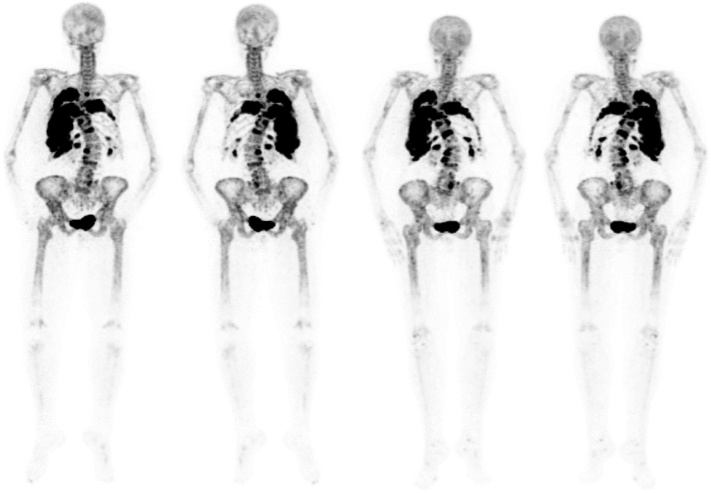
Na[18*F*]F PET-CT of case 2 before denosumab treatment (left, anterior and posterior view) and after 1.5 years of therapy (right, anterior and posterior view). Visually, the intensity appears similar at the second scan, mainly due to overprojection. Nevertheless a decrease in SUVpeak by 11% was measured, in SUVmean by 16%, FTV by 13% and TLF by 27%.

Radiographically the largest lesions in the right ribs decreased in both volume and intensity (Fig. 6): SUVpeak decreased by 11%, SUVmean by 16%, FTV by 13% and TLF by 27%.

**Fig. 7:**
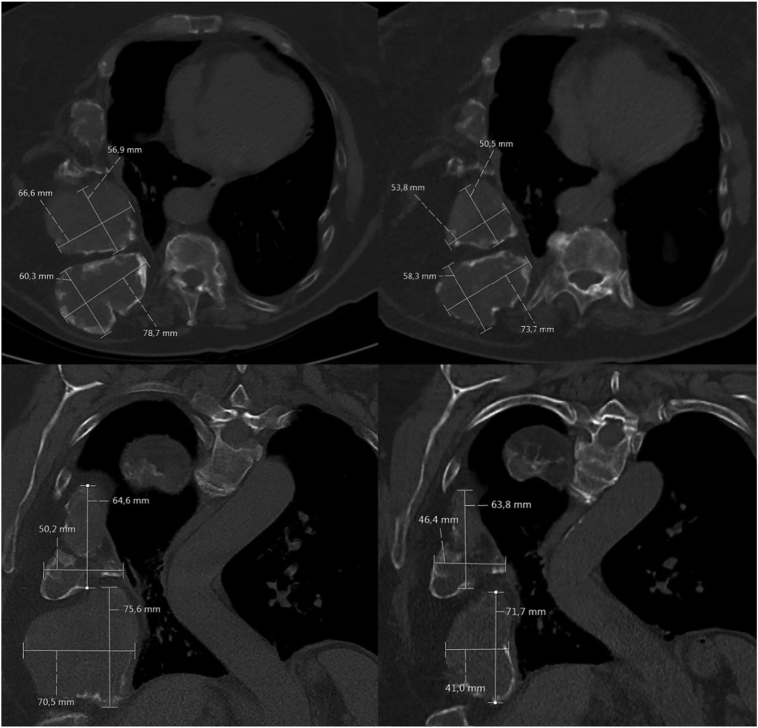
Regression of rib lesions before denosumab treatment (left, upper row: axial plane, lower row: coronal plane) and after 3.5 denosumab treatment (right: upper row: axial plane, lower row: coronal plane) on chest CT-scan of case 2.

On plain CT, the HU again ranged from 12 to 1180, although the volume of the lesions was mainly the result of lytic, cystic expansion, and to a lesser extent of fibrous tissue when compared to case 1. Also CT imaging revealed a decrease in size (Fig. 7), mainly caused by a decrease in the lytic/cystic components with HUs of 10–40.

This patient has now used denosumab 60 mg 3-montly for 5.3 years without side effects.

## 3 Discussion

These cases indicate that both size and activity of FD lesions detected by Na[^18^*F*]F PET-CT can decrease after treatment with denosumab, which occurred in both patients alongside an excellent clinical and biochemical response and persisted even after more than 5 years of treatment. This finding is relevant because it demonstrates the potential of denosumab therapy to induce radiographic changes on one hand and shows that Na[^18^*F*]F PET-CT is suitable for detecting lesional treatment response on the other hand. This denosumab-induced decrease in uptake and in size has been observed in a FD/MAS mouse model (Palmisano et al., 2019) but not in humans, where only a slower growth rate has been described (Boyce et al., 2012).

In the presented cases Dmab was started as last resort and off label. The off label prescription of medication is permitted in the Netherlands with regulatory compliance and with informed consent of the patient. The first results of the use of Dmab for FD/MAS in our center were published as part of a larger cohort (Majoor et al., 2019). These results however did not include radiological changes as these took longer to appear. Initially denosumab treatment was prescribed according to the regimen used in osteoporosis, but since the observed decrease in pain and biochemical response did not sustain throughout the 6 months, the patients were continued on a 3-montly schedule of 60 mg. Although a higher dose given more frequently, like in giant cell tumors of bone (Chawla et al., 2013), might induce more effects on lesion size, this may also increase the risk for serious side effects such as osteonecrosis of the jaw (ONJ) and atypical femur fractures (AFF). Prevention of and screening for side effects is part of our standard care trajectory for FD/MAS. Regarding ONJ, prior to starting denosumab a dental examination and appropriate preventive dentistry is performed. Dental procedures are conducted with antibiotics prophylaxis and with deferral of the next Dmab injection. Screening for AFFs is conducted on extended dual-energy X-ray absorptiometry (DXA), performed every year after 2 years of Dmab use, since early detection and management may prevent complete femur fractures (van de Laarschot et al., 2017).

Neoadjuvant use of denosumab may improve surgical possibilities and outcome and could be considered in severe FD cases. However it must be emphasized that denosumab treatment should only be considered as last resort, when bisphosphonate therapy is not effective or not tolerated, and when surgery is not (yet) possible, as no studies have investigated the effect of withdrawal and possible rebound phenomenon including hypercalcemia (Boyce et al., 2012). In our center patients with FD/MAS discontinuing Dmab therapy are scheduled for close clinical and biochemical monitoring every 3 months after withdrawal.

The two reported patients wish to continue their treatment (under close monitoring) despite the prolonged use of Dmab and the risk for side effects, because in their opinion the benefits in quality of life overshadow the potential disadvantages.

In our center patients who started on Dmab were evaluated with Na[^18^*F*]F PET-CT, an objective and quantitative measurement of which the parameters correlate with bone turnover markers (Papadakis et al., 2019; van der Bruggen et al., 2020) and skeletal outcomes such as fractures (Papadakis et al., 2019). Other benefits of Na[^18^*F*]F PET-CT include better pharmacokinetic characteristics and lower radiation burden compared to bone scintigraphy, 3D visualization, high resolution (Papadakis et al., 2019; van der Bruggen et al., 2020; Segall et al., 2010), and the potential to measure local treatment response to Dmab in FD/MAS (Van der Bruggen et al., 2021).

In conclusion, these cases illustrate the potential of denosumab therapy, in particular for lesions not responding to bisphosphonate therapy or not (yet) suitable for surgical treatment, and emphasize the importance of nuclear imaging to assess lesional response to denosumab treatment next to biochemical and clinical follow up, in particular when aiming to evaluate each lesion distinctively. Denosumab seems a promising drug but certain gaps in our knowledge require closure before denosumab can be widely applied for the treatment of FD/MAS.

## Declaration of competing interest

M.E. Meier was supported by a grant ‘Beter Bot’ from the 10.13039/501100005040Bontius Foundation, a nonprofit institution supporting research within the Leiden University Medical Center. No funding was provided by any pharmaceutical company. N.M.A.-D. received consulting fees for scientific advisory board meetings for Amgen, Netherlands. The other authors do not have any disclosures to report.
